# Associations of vertebral deformities and osteoarthritis with back pain among Japanese women: the Hizen-Oshima study

**DOI:** 10.1007/s00198-012-2038-2

**Published:** 2012-07-27

**Authors:** H. Kitahara, Z. Ye, K. Aoyagi, P. D. Ross, Y. Abe, S. Honda, M. Kanagae, S. Mizukami, Y. Kusano, M. Tomita, H. Shindo, M. Osaki

**Affiliations:** 1 Department of Orthopaedic Surgery, Nagasaki University Graduate School of Biomedical Sciences, Nagasaki, Japan; 2 Department of Public Health, Nagasaki University Graduate School of Biomedical Sciences, Nagasaki, Japan; 3 MedStrat Communications, Rahway, NJ USA; 4 Department of Rehabilitation, Nishi-Isahaya Hospital, Isahaya, Japan; 5 Department of Community Development, Nagasaki Wesleyan University, Isahaya, Japan

**Keywords:** Back pain, Epidemiology, Osteoporosis, Vertebral deformity, Vertebral osteoarthritis

## Abstract

**Summary:**

We examined the spinal distribution of the types of vertebral deformities and the associations of vertebral deformities and osteoarthritis with back pain in Japanese women. Midthoracic and upper lumbar vertebrae were more susceptible to deformity. Vertebral deformity and osteoarthritis were frequent and were associated with back pain.

**Introduction:**

Vertebral fractures due to osteoporosis and osteoarthritis are both common and significant health problems in aged people. However, little is known about the descriptive epidemiology of the individual deformity types and the relative clinical impact in women in Japan.

**Methods:**

Lateral radiographs were obtained from 584 Japanese women ages 40 to 89 years old. Deformities were defined as vertebral heights of more than 3 standard deviations (SDs) below the normal mean. Osteoarthritis was defined as Kellgren–Lawrence (KL) grade 2 or higher. Information on upper or low back pain during the previous month was collected by questionnaire. We compared the spinal distribution of the three types of vertebral deformities (wedge, endplate, and crush) typical of fractures and examined the associations of number and type of vertebral deformities and osteoarthritis with back pain.

**Results:**

Fifteen percent of women had at least one vertebral deformity and 74% had vertebral osteoarthritis. The prevalence of upper or low back pain was 30.1%. Deformities were most common in the midthoracic and upper lumbar regions and wedge was the frequent type, followed by endplate and crush. Multiple logistic regression analysis showed that the odds of back pain was 3.0 (95% CI 1.5–6.3) times higher for women with a single wedge deformity and 3.2 (95% CI 1.0-–0.6) times higher for women with two or more wedge deformities, compared to women with no wedge deformity. Vertebral osteoarthritis was associated with back pain (OR 1.8, 95% CI 1.1–2.9), independent of other covariates including age and deformities.

**Conclusion:**

Our results in this group of Japanese women are similar to and consistent with results reported previously in other populations of Japanese and Caucasians.

## Introduction

Osteoporotic fractures are significant health problems that impact health care costs and health-related quality of life of older people [1–3]. Vertebral fracture, the most frequent osteoporotic fracture, is an important harbinger of future vertebral and nonvertebral fracture independence of bone mineral density [4, 5]. Vertebral fractures occur in approximately 20 % of postmenopausal women [6–8], but two-thirds of vertebral fractures do not come to clinical attention [9, 10], perhaps because symptoms are absent or missed [11, 12]. Fractures are usually classified radiologically into one of three types of vertebral deformity (wedge, endplate, and crush) by measuring anterior, middle, and posterior vertebral heights. Although not all deformities are due to osteoporotic fracture, spatial distributions of the three types of vertebral deformity and the relationships of the number and type of deformity with clinical outcomes such as back pain may provide insights as to pathogenesis and consequences of vertebral fractures.

Previous studies conducted in western countries suggest that wedge is the most frequent type of vertebral deformity and that there is a peak occurrence in the midthoracic spine and around the thoraco-lumbar junction [6, 13–16]. Several studies reported associations between all three types of deformity and back pain [13, 17]. However, little is known about the descriptive epidemiology of the individual deformity types and the relative clinical impact in women in Japan.

Vertebral osteoarthritis is also common in elderly persons and is characterized by osteophytosis and disc degeneration [18, 19]. A cross-sectional study among men and women aged 50 years and over showed that 84 % of men and 74 % of women had at least one vertebral level with a grade 1 or higher osteophyte [18]. Several studies reported that vertebral osteoarthritis was associated with back pain [18, 20–23].

We previously reported that vertebral deformities were associated with back pain and physical disability in Japan and the US, and women with multiple vertebral deformities had significantly greater impaired function [24, 25]. However, relatively few studies have examined associations of type and location of vertebral deformity or osteoarthritis with location of back pain. Therefore, we conducted a cross-sectional study to characterize the distribution of the three types of vertebral deformity and examine the associations of number, type, and location of vertebral deformity and osteoarthritis with back pain in Japanese women. The focus of this study was on associations of vertebral deformities with back pain, but vertebral osteoarthritis was also analyzed in order to control for this potential confounding variable despite the difficulties inherent in measuring vertebral osteoarthritis.

## Subjects and methods

### Study sample

This was a cross-sectional analysis using baseline data of a prospective population-based cohort study. The subjects were Japanese women aged 40–89 years who participated in the Hizen-Oshima Study, a prospective population-based cohort study of musculoskeletal conditions (e.g., osteoporosis and osteoarthritis). We recruited community-dwelling women aged 40 years and over in Oshima, Nagasaki prefecture, Japan. The women were identified by the municipal electoral list and invited to participate through a single mailing. The town of Oshima has a population of approximately 5,800; all women aged 40 and over (*n* > 2,000) were invited to participate. The baseline examination was performed at the Oshima Health Center between 1998 and 1999, where height and weight measurements, questionnaires, and x-rays were conducted. A total of 586 women participated in the study. The mean age of participants (63.9 years) was significantly higher than that of nonparticipants (61.1 years). All participants were noninstitutionalized, living independently at baseline. This study was approved by the local ethics committee, and all subjects gave written informed consent before examination. Additional details of the Hizen-Oshima study have been previously published [25].

### Measurements

All participants were asked if they had back pain on most days during the previous month. The back pain questionnaire did not assess possible vertebral fracture date or duration of back pain. The location of back pain was asked separately: upper back (thoracic region) or low back (lumbar region). Information on the number of painful joints at nonspine sites was based on the subject’s responses to the following question: “which of your joints have ever been painful on most days during the previous 1 month?” Specific response categories (shoulders, elbows, wrists, hands and fingers, hips, knees, ankles, and feet) on both sides of the body were provided on an illustration of the skeleton. Height was measured without shoes using a wall-mounted stadiometer, and weight was measured with the subject in light clothing using a daily calibrated standard scale. Body mass index (BMI) was calculated as weight (kilogram)/height (meter)^2^.

### Spine radiographic assessment (vertebral deformities and osteoarthritis)

Lateral radiographs were obtained with the subject lying on her side with knees bent. All radiographs were obtained using a tube-to-film distance of 105 cm, with the tube positioned approximately over T-8 for thoracic films and L-2 for lumbar films.

#### Vertebral deformities

Radiographs were evaluated morphometrically by a single reader (KA). The anterior, medial, and posterior top and bottom of each vertebral body (T-4 to L-4) on the lateral films were marked on the film using a pencil. The anterior, medial, and posterior heights were measured with the aid of a microcomputer-linked caliper. Vertebral heights were measured on the thoracic film for thoracic vertebrae and on the lumbar film for lumbar vertebrae. The points indicating the border of the vertebral centrum were chosen based on the procedure described by Gallagher et al. [26] and Spencer et al. [27]. Radiographic vertebral deformities were defined as vertebral heights more than 3 SDs below the vertebra-specific population mean on the radiograph; vertebrae that met this posterior height criterion were classified as crush. The remaining vertebrae that had an anterior height reduction were called wedge. The remaining vertebrae that only had a central height reduction were called endplate. The timing of deformities could not be determined in this cross-sectional study.

#### Vertebral osteoarthritis

Radiographs were scored by a single reader (HK) for osteoarthritis of the thoracic spine in T4–T12 or lumbar spine in L1–L4 using the Kellgren–Lawrence (KL) grade as follows: KL0, normal; KL1, slight osteophytes; KL2, definite osteophytes; KL3, disc space narrowing with large osteophytes; and KL4, bone sclerosis, disc space narrowing, and large osteophytes [28]. In the present study, we defined the spine with disc space narrowing with and without osteophytes as KL3 [19]. KL grade was determined at intervertebral spaces, and the highest scores among thoracic or lumbar intervertebral spaces were then identified as the KL grade for that individual. Osteoarthritis was defined as KL grade 2 or higher. To evaluate the intrarater reliability of the KL grading, randomly selected radiographs of the thoracic and lumbar spine were scored by the same reader more than 1 month after the first reading for 40 individuals. The intrarater reliabilities were evaluated by kappa analysis. The reliability in KL grading of the thoracic or lumbar radiographs was found to be sufficient with kappa scores of 0.76 and 0.85, respectively. Radiographic readers (KA and HK) were blind to the subjects’ ages and other characteristics.

### Statistical analysis

For reasons of poor technical quality, the radiographs of two women did not allow reliable measurements of vertebral heights, leaving 584 women for the analyses. The Cochran–Armitage trend test was used to evaluate differences in the prevalence of back pain among age groups, and the chi-square test was used to evaluate differences among categories of number of vertebral deformities. Logistic regression analysis was used to explore the associations of type and number of vertebral deformity with back pain in the previous month; results are presented as odds ratios (ORs) with 95 % confidence intervals (CIs). Data analyses were performed with commercially available software (SAS Institute, Cary, NC).

## Results

The mean (SD) of age and BMI were 64.4 (9.6) years and 23.4 (3.5) kg/m^2^, respectively (Table 1). Fifteen percent of women had at least one vertebral deformity and 74 % had vertebral osteoarthritis. Forty-nine percent of women reported at least one painful joint at nonspine sites and 91 % were postmenopausal. The prevalence of upper back pain and low back pain were 19.2 % and 19.4 %, respectively (Table 2). The overall prevalence of upper or low back pain was 30.1 %, and differences among age groups were not significant overall (*p* = 0.32).

**Table 1:** Basic characteristics of study subjects (*N* = 584)

Variable	
	Mean (SD)
Age (years)	64.4 (9.6)
Height (cm)	149.7 (6.1)
Weight (kg)	52.4 (8.9)
Body mass index (kg/m^2^)	23.4 (3.5)
	Number (%)
Women with at least one vertebral deformity	86 (14.7 %)
Women with vertebral osteoarthritis	431 (73.8 %)
Women with at least one painful joint at nonspine site	283/575 (49.2 %)^a^
Postmenopausal	530 (90.8 %)

^a^Data is missing for some individuals, but denominator is given

**Table 2:** The prevalence of women with back pain in the previous 1 month according to age

Age group (years)	No. of subjects	Upper back pain (no. (%))	Low back pain (no. (%))	Upper or low back pain (no. (%))
40–49	45	6 (13.3)	7 (15.6)	11 (24.4)
50–59	123	23 (18.7)	27 (22.0)	40 (32.5)
60–69	217	36 (16.6)	39 (18.0)	58 (26.7)
70–79	169	39 (23.1)	32 (18.9)	56 (33.1)
80–89	30	8 (26.7)	8 (26.7)	11 (36.7)
Total	584	112 (19.2)	113 (19.4)	176 (30.1)
		*P* = 0.08^a^	*P* = 0.68^a^	*P* = 0.32^a^

^a^Cochran–Armitage trend test

Table 3 presents the frequency distribution of the three types of deformity and back pain. The majority of deformities were wedge, followed by endplate and crush. In univariate analysis, thoracic deformities were not associated with upper back pain, but lumbar wedge and endplate deformities were significantly associated with low back pain. Overall, wedge and endplate deformities were associated with any (upper or low) back pain.

**Table 3:** Frequency (%) and distribution of type of vertebral deformity and back pain (*n* = 584)

Deformity type	No. of deformities	Location	Pain
		Thoracic	Upper back
Wedge	0	566 (96.9)	109/566 (19.3)
	1	18 (3.1)	3/18 (16.7)
	2+	0 (0.0)	–
			*P* = 0.78^a^
Endplate	0	574 (98.3)	109/574 (19.0)
	1	8 (1.4)	3/8 (37.5)
	2+	2 (0.3)	0/2 (0.0)
			*P* = 0.33^a^
Crush	0	574 (98.3)	110/574 (19.2)
	1	5 (0.9)	0/5 (0.0)
	2+	5 (0.9)	2/5 (40.0)
			*P* = 0.27^a^
Any	0	549 (94.0)	104/549 (18.9)
	1	26 (4.5)	6/26 (23.1)
	2+	9 (1.5)	2/9 (22.2)
			*P* = 0.85^a^
		Lumbar	Low back
Wedge	0	557 (95.4)	99/557 (17.8)
	1	21 (3.6)	9/21 (42.9)
	2+	6 (1.0)	5/6 (83.3)
			*P* < 0.0001^a^
Endplate	0	561 (96.1)	103/561 (18.4)
	1	16 (2.7)	4/16 (25.0)
	2+	7 (1.2)	6/7 (85.7)
			*P* < 0.0001^a^
Crush	0	574 (98.3)	109/574 (19.0)
	1	7 (1.2)	2/7 (28.6)
	2+	3 (0.5)	2/3 (66.7)
			*P* = 0.094^a^
Any	0	534 (91.4)	92/534 (17.2)
	1	32 (5.5)	8/32 (25.0)
	2+	18 (3.1)	13/18 (72.2)
			*P* < 0.0001^a^
		Total	Upper or low back
Wedge	0	524 (89.7)	145/524 (27.7)
	1	43 (7.4)	20/43 (46.5)
	2+	17 (2.9)	11/17 (64.7)
			*P* = 0.0002^a^
Endplate	0	543 (93.0)	156/543 (28.7)
	1	23 (3.9)	9/23 (39.1)
	2+	18 (3.1)	11/18 (61.1)
			*P* = 0.0082^a^
Crush	0	562 (96.2)	167/562 (29.7)
	1	13 (2.2)	5/13 (38.5)
	2+	9 (1.5)	4/9 (44.4)
			*P* = 0.51^a^
Any	0	498 (85.3)	136/498 (27.3)
	1	44 (7.5)	18/44 (40.9)
	2+	42 (7.2)	22/42 (52.4)
			*P* = 0.0013^a^

^a^Chi-square test

Table 4 presents the frequency distribution of the different combinations of vertebral deformity types. About 68 % of subjects with vertebral deformity had only one type of deformity type present, and wedge only (36.8 %) was the most frequent type followed by endplate only (21.8 %) and crush only (9.2 %). Among subjects with more than one type of deformity, wedge and endplate (16.1 %) were the most frequent types followed by three types of deformity (9.2 %), wedge, and crush (6.9 %).

**Table 4:** The frequency distribution of combinations of vertebral deformity types

Type of vertebral deformity	No. (%) of women with vertebral deformity
Wedge only (%)	32 (36.8)
Endplate only (%)	19 (21.8)
Crush only (%)	8 (9.2)
Wedge and endplate (%)	14 (16.1)
Wedge and crush (%)	6 (6.9)
Endplate and crush (%)	0 (0.0)
All three types of deformity (%)	8 (9.2)

**Fig. 1:**
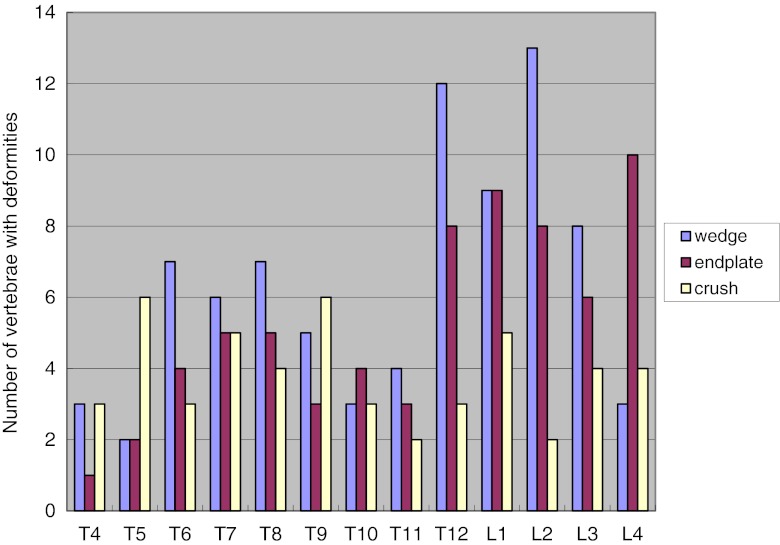
Number of vertebral deformities by type and vertebral level. The number of deformities was highest in the T12–L4 region with a smaller peak centered at T7–T8. Wedge was the most frequent type of deformity and showed a predilection for the thoraco-lumbar region (T12–L3). Endplate deformity showed a predilection from T12 to L4. Crush deformity was less frequent and showed no predilection for anatomical location

In univariate analyses (Table 5), thoracic and lumbar vertebral osteoarthritis were not significantly associated with upper or low back pain, respectively. Overall, vertebral osteoarthritis was significantly associated with any (upper or low) back pain (*p* = 0.013). Figure 1 shows the anatomical distribution of vertebral deformities. The number of deformities was highest in the T12–L4 region with a smaller peak centered at T7–T8. Wedge was the most frequent type of deformity and showed a predilection for the thoraco-lumbar region (T12–L3). Endplate deformity showed a predilection from T12 to L4. Crush deformity was less frequent and showed no predilection for anatomical location.

**Table 5:** Frequency (%) of vertebral osteoarthritis and back pain (*n* = 584)

Vertebral osteoarthritis	Location	Pain
	Thoracic	Upper back
Without	221 (37.8)	37/221 (16.7)
With	363 (62.2)	75/363 (20.7)
		*P* = 0.24^a^
	Lumbar	Low back
Without	309 (52.9)	52/309 (16.8)
With	275 (47.1)	61/275 (22.2)
		*P* = 0.10^a^
	Total	Upper or low back
Without	153 (26.2)	34/153 (22.2)
With	431 (73.8)	142/431 (33.0)
		*P* = 0.013^a^

^a^Chi-square test

In 15 separate age-adjusted logistic regression models, no significant associations were observed between types of thoracic deformities or osteoarthritis and upper back pain (Table 6). Significant associations with low back pain were observed for wedge, multiple endplate, and multiple deformities in lumbar vertebrae. Moreover, the associations between lumbar deformities (especially multiple deformities) and low back pain tended to be much higher than the associations between thoracic deformities and upper back pain. The odds of any (upper or low) back pain was 2.4 (95 % CI: 1.2–4.5) times higher for women with a single wedge deformity and 5.2 (95 % CI: 1.8–14.8) times higher for women with two or more wedge deformities, compared to women with no wedge deformity. In separate analyses of endplate and crush deformities, there were no significant associations except for two or more endplate deformities. Analysis combining all types of deformities showed both a single deformity of any type (OR 1.9, 95 % CI 1.0–3.6) and two or more deformities (OR 2.9, 95 % CI 1.5–5.7) were significantly associated with any (upper or low) back pain, independent of age. The odds of any (upper or low) back pain was 1.7 (95 % CI 1.1–2.6) times higher for women with vertebral osteoarthritis (at any location), compared to women without osteoarthritis, independent of age.

**Table 6:** Age-adjusted association of type and number of vertebral deformities or osteoarthritis with back pain in the previous month

		Thoracic vertebrae vs. upper back pain		Lumbar vertebrae vs. low back pain		Total vertebrae vs. upper or low back pain	
Type	No.	Odds ratio	95 % confidence interval	Odds ratio	95 % confidence interval	Odds ratio	95 % confidence interval
Wedge	0	1.0	–	1.0	–	1.0	–
	1	0.7	0.2–2.6	3.8	1.5–9.6	2.4	1.2–4.5
	2+	–	–	26.4	3.0–234.5	5.2	1.8–14.8
Endplate	0	1.0	–	1.0	–	1.0	–
	1	2.3	0.5–9.7	1.5	0.5–4.9	1.6	0.7–3.8
	2+	–	–	27.2	3.2–231.6	3.8	1.4–10.3
Crush	0	1.0	–	1.0	–	1.0	–
	1	–	–	1.7	0.3–8.8	1.4	0.5–4.4
	2+	2.5	0.4–15.3	8.3	0.7–93.0	1.8	0.5–6.8
Any	0	1.0	–	1.0	–	1.0	–
	1	1.1	0.4–2.9	1.8	08–4.3	1.9	1.0–3.6
	2+	1.0	0.2–5.2	14.5	4.8–43.4	2.9	1.5–5.7
Osteoarthritis	Without	1.0	–	1.0	–	1.0	–
	With	1.2	0.8–1.9	1.4	0.9–2.2	1.7	1.1–2.6

There were 15 separate analyses; age was included as a continuous covariate in each model

Including vertebral deformities and osteoarthritis together with additional adjustment for BMI, number of painful nonspine joints (ordinal), and numbers of other types of vertebral deformity (ordinal) did not substantially alter these results (Table 7).The odds of upper or low back pain was 3.0 (95 % CI 1.5–6.3) times higher for women with a single wedge deformity, and 3.2 (95 % CI 1.0–10.6) times higher for women with two or more wedge deformities, compared to women with no wedge deformity. Total vertebral osteoarthritis was associated with upper or low back pain, independent of age, BMI, number of painful nonspine joints (ordinal), and vertebral deformity(OR 1.8, 95 % CI 1.1–2.9). We repeated the analyses using a definition of vertebral deformity based upon a 2 SD threshold instead of 3 SD in order to include the effect of milder deformities; similar results were obtained.

**Table 7:** Multiple adjusted association of type and number of vertebral deformities or osteoarthritis with back pain in the previous month

		Thoracic vertebral deformity or osteoarthritis vs. upper back pain		Lumbar vertebral deformity or osteoarthritis vs. low back pain		Total vertebral deformity or osteoarthritis vs. upper or low back pain	
Type	No.	Odds ratio	95 % confidence interval	Odds ratio	95 % confidence interval	Odds ratio	95 % confidence interval
Model 1							
Wedge	1	0.7	0.2–2.9	2.7	1.0–7.9	3.0	1.5–6.3
	2+	–	–	15.9	1.5–162.7	3.2	1.0–10.6
Osteoarthritis	With	1.4	0.9–2.2	1.4	0.8–2.2	1.8	1.1–2.9
Model 2							
Endplate	1	2.7	0.6–12.1	1.5	0.4–5.8	1.0	0.3–2.7
	2+	–	–	16.7	1.8–154.0	3.0	0.9–10.1
Osteoarthritis	With	1.4	0.9–2.2	1.4	0.8–2.2	1.8	1.1–2.9
Model 3							
Crush	1	–	–	1.1	0.2–7.4	0.7	0.2–2.6
	2+	3.9	0.6–25.5	3.9	0.3–47.4	0.9	0.2–4.3
Osteoarthritis	With	1.4	0.9–2.2	1.4	0.8–2.2	1.8	1.1–2.8
Model 4							
Any	1	1.0	0.3–3.0	1.9	0.8–4.6	2.3	1.2–4.5
	2+	1.3	0.3–6.6	11.1	3.5–35.0	2.8	1.4–5.8
Osteoarthritis	With	1.4	0.9–2.2	1.4	0.8–2.2	1.8	1.1–2.9

Each model was run three separate times (once each for upper, lower, and any (upper or lower) back pain) for a total of 15 separate analyses, each with covariates for age (continuous), body mass index (continuous), and number of painful nonspine joints (ordinal). There were four regression models; the model for Wedge deformity and osteoarthritis (Model 1) included ordinal variables for number of endplate and number of crush deformities; the model for Endplate deformity and osteoarthritis (Model 2) included ordinal variables for number of wedge and number of crush deformities; and the model for Crush deformity and osteoarthritis (Model 3) included ordinal variables for number of wedge and number of endplate deformities. The model for Any deformity and osteoarthritis (Model 4) did not include ordinal variables for numbers of other vertebral deformity types

## Discussion

We examined the prevalence of the three types of vertebral deformity by anatomic location and the associations of number and type of vertebral deformity or osteoarthritis with back pain among women in Japan. The prevalence of vertebral deformity was higher in the midthoracic and upper lumbar spine. Wedge deformity was the most frequent deformity type, with a predilection for the thoraco-lumbar region (T12–L3). Crush deformity was less frequent and showed no predilection for anatomical location. Significant associations with back pain were observed for wedge deformities, for vertebral deformities in general (in models that included all types) and for vertebral osteoarthritis.

Our results confirm findings from other population-based studies in women that wedge was the most frequent type of deformity [6, 13], and that the prevalence of deformity was higher in midthoracic and upper lumbar vertebrae [13, 15]. This distribution is believed to be related to biomechanical factors [29, 30]. Movements such as stooping or lifting greatly increase loading on the spine, especially the midthoracic and upper lumbar vertebrae where the spine curves. Furthermore, the thoraco-lumbar junction consists of an articulation between the relatively rigid thoracic spine and the freely mobile lumbar segments, maximizing compression stresses. Consequently, some experts recommend that patients with osteoporosis avoid certain movements and activities that increase load on the spine to reduce the risk of vertebral fractures.

Our cross-sectional findings are consistent with previous reports that all three types of deformity were associated with back pain [13, 17], although wedge was the only specific type of deformity that was significant in our study. One possibility is that, among these Japanese women, wedge deformities may be more strongly associated with back pain than endplate or crush deformities because wedge deformity increases kyphosis, contributing to increased paravertebral muscle strain or back pain. Such effects on spinal curvature might contribute to back pain long after the acute fracture pain has subsided. Another possibility is that the smaller numbers of endplate and crush deformities may have reduced the statistical power to detect significant associations. Indeed, the odds of back pain were increased for endplate and crush deformities but did not attain significance in most cases.

In our study, the odds of back pain increased with the number of wedge deformities. Ettinger et al. [17] reported similar results, showing that multiple severe deformities tended to be associated with increased back pain. Furthermore, prospective studies showed that the risk of back pain increased with the number of incident vertebral fractures [31, 32].

In prospective studies of both clinical and morphometric vertebral fractures, back pain was associated with incident vertebral fracture [31–33]. It is likely that the cross-sectional associations reported here underestimate the impact of acute vertebral fractures on back pain; previous prospective studies have shown that new vertebral fractures have stronger associations with pain than do existing deformities identified in cross-sectional analyses [32, 34].

We also found a significant association of vertebral osteoarthritis with any (upper or low) back pain. Previous studies showed that lumbar vertebral osteoarthritis was associated with low back pain [20–23]. In our analysis, the association of lumbar osteoarthritis with low back pain was not statistically significant after adjusting for age, perhaps because of limited statistical power.

In our analysis, lumbar deformity was significantly associated with lumbar back pain, but thoracic deformities were not significantly associated with upper back pain. As others have noted, the rib cage may help stabilize the thoracic spine, thereby reducing pain associated with deformities, whereas the lumbar spine is more flexible and less stable, which may increase loads on paravertebral muscles and contribute to back pain.

Our study had some limitations. Because this was a cross-sectional setting, a causal relationship was not necessarily demonstrated by our results. Only ~30 % of eligible women participated in this study, which is a potential source of selection bias. The women who participated in the study were younger on average than the general population. Women with more symptoms may have chosen to participate. Alternatively, women with the most severe deformities or the most severe symptoms and disability may have chosen not to participate because they had to be mobile enough to attend the study site. Other clinical outcomes of vertebral deformity such as height loss or kyphosis were not available for analysis in our study. Because this study only included women, our findings may not be generalizable to men.

In conclusion, our results are consistent with other population-based studies that reported vertebral deformities are most common in midthoracic and upper lumbar vertebrae and suggest that the number and type of vertebral deformities and osteoarthritis are important sources of back pain among women in Japan. Although these findings are subject to limitations that are typical of cross-sectional studies, they are broadly consistent with results from other studies of Japanese and Caucasians that used prospective and cross-sectional designs.
